# Endoscopic dacryocystrhinostomy

**DOI:** 10.1016/S1808-8694(15)31238-6

**Published:** 2015-10-20

**Authors:** Bernardo Cunha Araujo Filho, Richard Louis Voegels, Ossamu Butugan, Carlos Diogenes Pinheiro Neto, Marcus Miranda Lessa

**Affiliations:** Ph.D. studies under course, Division of Clinical Otorhinolaryngology, HCFMUSP, Specialist in Otorhinolaryngology, SBORL; Associate Professor, Discipline of Otorhinolaryngology, Medical School, University of Sao Paulo, Director of Rhinology, HCFMUSP; Full Professor, Discipline of Otorhinolaryngology, Medical School, University of Sao Paulo; Resident Physician, Division of Clinical Otorhinolaryngology, Hospital das Clínicas, Medical School, University of Sao Paulo; Ph.D. in Otorhinolaryngology, HCFMUSP, Professor of Otorhinolaryngology, Faculdade Federal da Bahia. Division of Clinical Otorhinolaryngology, Hospital das Clínicas, Medical School, University of Sao Paulo

**Keywords:** dacryocystrhinostomy, endoscopic, endonasal, dacryocystitis

## Abstract

Endonasal endoscopic dacryocystorhinostomy (EN-DCR) is now a well-established procedure to relieve nasolacrimal duct obstruction, becoming its domain for the ENT surgeons indispensable.

**Aim:**

The aim of the present study is to report the experience of the Otorhinolaryngology Department of the University of São Paulo Medical School in the management of the obstruction of the drainage of the nasolacrimal system by EN-DCR, comparing with the results in literature.

**Study design:**

clinical retrospective.

**Material and Method:**

We reviewed the medical records of 17 patients (17 eyes) that were submitted to EN-DCR between april 2001 and july 2004. We analysed: sex, age at the time of diagnosis, etiology, clinical findings, surgical technique, use of silicone tubes, follow-up and complications.

**Results:**

Eight men and nine women, the age range was from 29 to 79 years (mean 42.6413.1 years), mean follow-up time: 15 months, presented a lacrimal clinic with epiphora. Powered DCR was performed in 06 cases and YAG LASER in 01 patient. Silicone tubes were used in all cases and left in place mean 7.9 weeks. The surgical success rate was 82,3%.

**Conclusion:**

EN-DCR showed one safe technique, with advantages in relation to the external technique. So ophthalmologists and ENT physicians must work in harmony to offer more benefits to its patients.

## INTRODUCTION

Infection of the lachrymal pathways has attracted the attention of physicians for some time, however, as a result of the development of antibiotics, acute forms do not represent a life-threatening condition as they used to in the past, and at the same time the chronic forms became more prevalent.

The history of lacrimal pathway surgery dates back from Hamurabi (2,200 B.C.)^1^. Since then, techniques for treatment of lacrimal pathways have been developed, fighting against infections and restoring the transit of tears through the lacrimal system. Thus, dacryocystorhinostomy (DCR) has been the treatment of choice for cases of distal obstruction of lacrimal system (below the common canaliculi) and consists in creating an anastomosis between the lacrimal sac and the basal cavity, enabling the tear to drain to the lower meatus, relieving the symptoms.

Technological innovations and surgeries of lacrimal system that are less invasive were developed to reduce morbidity and to improve the results. Traditionally, the treatment of nasolacrimal obstruction is external DCR, frequently performed by ophthalmologists. This technique was described by Toti in 1904 and modified by Dupuy-Dutemps and Bourguet^2^, with the suture of mucous flaps.

The endonasal approach was described for the first time by Caldwell in 1893^3^, but it was forgotten for decades by the limited vision and the assessment of nasosinusal anatomy. The introduction of microscope and later the endoscopic techniques, associated with the close relation of lacrimal systems and the nasal fossa, have made endonasal surgical treatment of low lacrimal affections very popular among Otorhinolaryngologists^4^. Currently, endoscopic DCR is a well-accepted and established technique in the treatment of obstruction of the lacrimal sac and nasolacrimal duct^1^^,^^3^^,^^5^ and its proficiency by Otorhinolaryngologists has become indispensable.

The purpose of the present article is to present the experience in endoscopic DCR of the Division of Clinical Otorhinolaryngology, HCFMUSP, comparing and discussing our results with those found in the literature.

## MATERIAL AND METHODS

This study was based on the retrospective analysis of 17 medical charts of patients submitted to endoscopic DCR, admitted to the Division of Clinical Otorhinolaryngology, Hospital das Clínicas, Medical School, University of Sao Paulo, in the period between April 2001 and July 2004. Out of 17 patients (17 eyes), 8 were men (47.1%) and 9 were women (52.9%). The age at diagnosis ranged from 29 to 79 years, with mean age of 42.64 years and standard deviation of 13.1 years.

Patients were assessed according to:

a. Gender
b. Age at time of surgery
c. Etiology
d. Clinical presentation
e. Surgical technique performed (cold or laser instrument)
f. Use of silicone tube and duration of insertion
g. Number of surgeries necessary to correct
h. Some aspects of postoperative follow-up, such as the use of antibiotics and the observed complications.

The results were statistically analyzed with Fischer exact test and significance level of p < 0.05.

### Surgical technique

Procedures were performed under general anesthesia, but in one case (LASER YAG) we used local anesthesia with sedation. We preferred general anesthesia owing to greater comfort of the patients and possible correction of septal deviations, bullous and paradoxical conchae, if necessary. After topical vasoconstriction of nasal cavity with lidocaine solution at 2% with adrenaline 1:2,000, we infiltrated lidocaine 2% with adrenaline at 1:80,000 in the anterior region to the medium concha. We used 0 degree and 4mm endoscopes (Hopkins-Karl Storz). Under endoscopic visualization, we made a mucous rectangular flap with the posterior base adjacent to the medium concha, 1 cm2 subperiosteal using Sickle Knife and detacher-aspirator or Cottle. We positioned the flap posteriorly during the procedure, protecting the anterior insertion of the medium concha against traumas. After exposure of lacrimal bone and frontal process of maxilla, we created a posterior window to access the sac and expanded the window anteriorly to expose the whole width of the lacrimal sac. In 6 patients we used power-driven instruments (motor and diamond bur) and in one case we used LASER YAG. We also used cold instruments (such as chisel and hammer) to prepare a rhinostome.

The lower limit of the bone window was the insertion of the lower concha on the nasal wall. The identification of the sac was made with a probe (Bowman) and through the canaliculi, it penetrated into the sac and was pushed medially, but it could also in some occasions be visualized by digital compression of the ocular medial cantus. It is advocated to remove the whole medial wall of nasolacrimal sac with clamping or Sickle Knife. Finally we cut a U-shaped mucous flap with anterior concavity to promote first intention healing, preventing bone exposure and less formation of granulomas.

A silicone tube was passed through the superior and inferior canaliculi up to nasal fossa and fixed in the vestibule, using nylon thread 3–0, maintaining the opening of the lacrimal pathways with the nasal fossa. We did not use any type of nasal packing. Frequent irrigation of the canaliculi with sterile solution was performed at outpatient level.

## RESULTS

Epiphora was the predominant complaint in all cases. The main complaint of lacrimal obstruction was idiopathic (58.8%), followed by septal surgeries and nasal conchae (17%) and trauma (11.2%). Synechia caused by transphenoid access for exeresis of macroadenoma of hypophysis and paranasal sinuses functional surgery were responsible for 2 cases. The mean time of follow-up was 15 months (minimum: 5 months and maximum: 37 months) and in all of the cases, we used silicone tubes for an average period of 7.9 weeks.

Four patients had been submitted to external DCR and were submitted to endoscopic procedure by obstruction of rhinostome. In one of these cases, septal deviation was responsible for the failure. Considering as surgical success the resolution of epiphora, the success of the endonasal technique was achieved in 82.3% of the cases. We observed that in one of the failures, we had employed LASER YAG. All recurrences have progressed well after the new endoscopic procedure up to the present moment.

Systemic antibiotics, such as first generation cephalosporins and topical ophthalmic drugs were used in the postoperative period, as well as frequent nasal lavage with sterile solution.

## DISCUSSION

In this study, we analyzed 17 patients who presented epiphora in a period of 3 years. The largest series found in the literature was described by Sprekelsen et al.^6^ in 1996, with 152 eyes submitted to DCR, followed by Yang et al.^7^. However, the criteria for therapeutic success and the postoperative follow-up are varied, hindering the comparison of results among the many different authors.

The stenosis of lacrimal paths, idiopathic in two thirds of the cases 1, family predisposition, anatomical variations of the duct and recurrent infection have been considered as possible causes. Our sample presented 58.2% of the cases caused by idiopathic stenosis, presenting a value close to the one described in the literature. Similarly to what was observed in our study, chronic sinusitis of maxillary and ethmoidal sinuses, septal deviations and acute rhinitis may cause ascending infection of the duct, resulting in inflammatory reaction, edema, ulceration and finally, chronic dacryocystitis. Bilateral stenosis is rare^1^^,^^8^, as observed by us.

Only one case was treated with LASER and the result was poor. The endoscopic approach with LASER was proposed to improving hemostasis in the endoscopic surgery and to reduce the formation of granulation tissue. However, despite these advantages, its high price and low effectiveness (64% to 85%), especially owing to the small ostium produced, has limited use to the technique^1^^,^^9^^,^^10^.

Cokkeser et al.^3^ advocated the use of chisel and hammer because it is a simpler, more hemostatic, cheaper and practical method to make a bone window. Conversely, Wormald et al.^11^, Sham et al.^12^ and Hartikainen et al.^2^ used drills to cause saccular exposure and ensure that through these instruments there is exposure of the more reliable lacrimal sac, which is ensured by the use of the chisel. Using the endonasal technique with chisel and hammer, and the use of power-driven drills, we did not observe any significant difference between these two approaches (p ≤ 0.05). Our results were similar to the findings in the literature.

We observed that endonasal access provided easy access to repair affections that could impair the results, such as septal deviations, bullous concha, synechia, such as observed in patients submitted to previous external DCR, a very flexible method to endonasal anatomical affections.

Regardless of the methods used, it is known that, despite the controversy, a wide bone window and exposure of the whole sac is determinant in surgical success. Cokkerser et al.^3^, Sham et al.^12^, Wormald et al.^13^ consider that the removal of the whole bone from the sac is associated with better index of success and visualization of opening of common canaliculi, after the removal of the media wall of the sac, which is a good indicator of appropriate saccular exposure^11^.

Lindberg et al.^14^, in their study, observed that the final size of rhinostome did not have any relation with the size of the bone window, however Heher et al.^15^, based on this assumption, managed to have a rate of only 70% success. Confirming the reported data, Welham et al.^16^, reviewed 205 failed DCR and concluded that the main cause of failure was incomplete opening of rhinostome and Millman et al.^17^ observed that fibrotic sacs presented therapeutic failure in 29%, whereas sacs with mucoceles had 82% failure.

At the end of the surgical procedure we may apply mitomycin C, preventing proliferation of scarring tissue^1^^,^^18^^,^^19^ and also mucous flap sutures. In our sample, we did not use these methods, but the use of silicone stents to prevent stenosis of rhinostome was used in all patients. Silicone tubes are used to maintain the opening of the ostium, and normally they are placed for a varied period of time, according to the authors' preference. Similarly to our study, Kong et al.^10^ advocated a maximum of 8 weeks. Unlu et al.^20^, comparing the groups with and without intubation with silicone after endoscopic DCR did not find significant results between the two groups (92% versus 87%, respectively). However, the level of complications was greater in the group of patients with intubation, despite the fact that Walland et al.^21^ reported that the use of silicone tubes did not increase the risk of stenosis.

Broad-spectrum systemic antibiotics and topical ophthalmologic drugs are used postoperatively. Similarly to the use of antibiotics, frequent daily nasal lavage with sterile solution and irrigation of lacrimal paths are essential in therapeutic success.

The DCR EN results ranged from 70% to 95% and depend on a series of factors, already reported. Cokkeser et al.^3^, comparing DCR EX to DCR EN in 115 patients had success rates of 89.8% and 88.2%, respectively, and recommend the use of endonasal techniques owing to their advantages comparing to the external approach. The success of the endonasal technique in our service was 82.3%, similarly to the results reported in the literature.

Many studies reported similar results or slight nonsignificant disadvantage of DCR EN in comparison to DCR EX. Hartikainem et al.^2^ compared these two techniques in 64 cases and found success rates in 75% for DCR EN and 91% for DCR EX. These poor results may be explained by the fact that these patients have been submitted to small rhinostomies, with 7mm diameter, emphasizing the importance of a wide window between the sac and the nasal cavity.

The main complication is therapeutic failure 16. In three cases, we did not succeed in the first endonasal approach, but all recurrences progressed well after the new endoscopic procedure. The reasons for failure may be owed to many different factors: excessive bleeding, wrong location of lacrimal sac, incision of inappropriate mucosa (leading to synechia and excessive granulation), failure in bone opening, bone neoformation, canalicular obstruction, SUMP syndrome and affections inherent to the nasal fossa (narrow nasal fossa, mucocele, etc). Other complications are: soft tissue infection after surgery (8%), bleeding, orbital complications, such as fat prolapse, corneal irritation, and nasal discomfort caused by silicone tubes^1^^,^^4^^,^^10^^,^^19^. Christmas et al.^22^ reported the complication of DCR EM that, owing to formation of synechia, caused blocked drainage of middle meatus, leading to frontal-ethmoidal sinusitis, a fact that should warn the Otorhinolaryngologists about the possible complications inherent to the procedure.

**Table 1:** Review of the literature about endonasal dacryocystorhinostomy.

Authors	Year	Number of eyes	Rate of success	Follow up	Comments
Sprekelsen et al.^6^	1996	152	96%	12 months	
Yang et al.^7^	1998	150	90%	13 months	Used uncinate process as the main repair sac
Hartikainem et al.^2^	1998	32	75%	12 months	Rhinostomy presented maximum diameter of 7mm (facilitating re-obstruction)
Minasian et al.^23^	1999	16	81%	6 months	5 failures by synechiae
Sham et al.^12^	2000	17	88%	13 months	Failure caused by orbital fat prolapse and canaliculi obstruction
Cokkeser et al.^3^	2000	51	88,2%	25 months	Used silicone tube for 2 to 3 months. Failure by learning curve in using the electrical elements (drill) to access NL sac
Wormald et al.^11^	2002	47	95,7%	11 months	
Unlu et al. ^20^	2002	14	85,7%	15 months	Compared DCR EN w/ and w/out silicone tube (did not find significant difference)
		(c/tubo)			
		6 (s/tubo)	81,3%		
HCFMUSP	2004	17	82,3%	15 months	Used silicone tube on average for 7.9 weeks.

## CONCLUSION

Endoscopic technique is a safe technique with promising results that provide a series of advantages compared to the external technique, such as avoiding scarring of the face and disarrangement of the medial canthal anatomy.

The two techniques, external and endoscopic, in experienced hands, present similar results and ophthalmologists and otorhinolaryngologists should work in harmony to provide more benefits to their patients.
